# Etanercept-Induced Pityriasis Lichenoides Chronica in a Patient with Rheumatoid Arthritis

**DOI:** 10.1155/2015/168063

**Published:** 2015-02-18

**Authors:** Andrés F. Echeverri, Andrés Vidal, Carlos A. Cañas, Andrés Agualimpia, Gabriel J. Tobón, Fabio Bonilla-Abadía

**Affiliations:** ^1^Rheumatology Unit, Fundación Valle del Lili, Carrera 98 N. 18-49, Cali, Colombia; ^2^Dermatology Unit, Fundación Valle del Lili, Carrera 98 N. 18-49, Cali, Colombia

## Abstract

We present a 74-year-old female patient who developed a pityriasis lichenoides chronica (PLC) during etanercept therapy. This association is not described in the literature and might be considered in the spectrum of cutaneous adverse reactions of etanercept.

## 1. Introduction

Pityriasis lichenoides chronica (PLC) is a rare inflammatory skin disorder of unknown etiology that is considered as a subtype of pityriasis lichenoides. Patients with PLC usually present with widely distributed red-brown papules on the trunk and extremities. The diagnosis of PLC is based upon the findings on the physical examination and a skin biopsy is required to confirm the diagnosis [1]. Several case reports have shown the association of PLC in patients with inflammatory diseases treated with antitumor necrosis factor alpha (anti-TNF*α*) therapies. [2–4]. To our knowledge this is the first case reported in the literature of PLC associated with etanercept treatment.

## 2. Case Report

**Figure 1:**
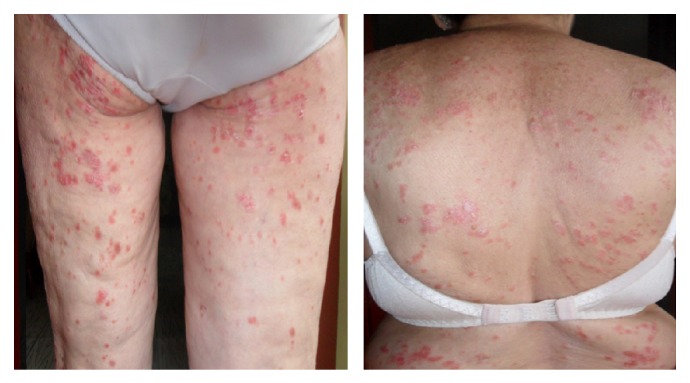
Erythematosus and desquamative papules with central scaling on the back and arms.

A 74-year-old female patient with seropositive rheumatoid arthritis (RA) diagnosed 18 years ago, with involvement in proximal interphalangeal joints, metacarpophalangeal, wrists, elbows, ankles, and left knee, was treated with chloroquine, prednisolone, and methotrexate for several years showing remission of the disease. In recent years, she presented an inflammatory flare with reappearance of articular inflammatory signs, elevated acute phase reactants, and functional limitation. For this reason leflunomide was added to treatment without clinical efficacy. Thus, biological treatment with etanercept 50 mg subcutaneous every week was started with improvement of inflammatory signs and symptoms. After six weeks of etanercept onset, she was evaluated by Rheumatology and Dermatology services for the appearance of erythematosus and desquamative papules, with central scaling on the back and arms (Figure 1). She had no personal or family history of psoriasis or other skin diseases. A skin biopsy was indicated.

**Figure 2:**
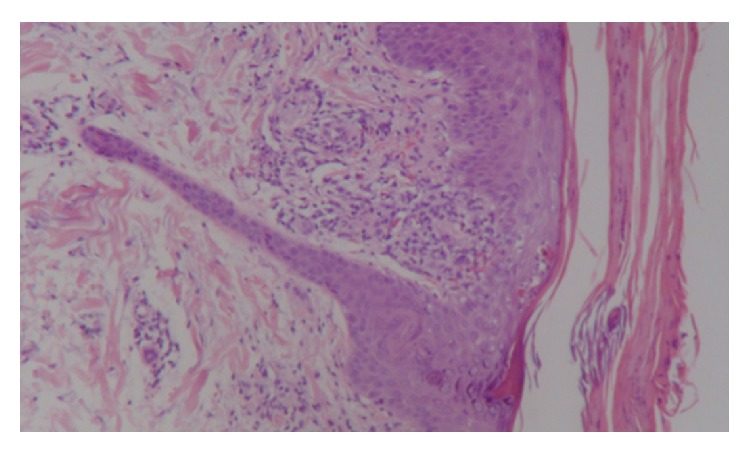
Pathology image of epidermis with parakeratosis and mild acanthosis and papillary dermis with perivascular lymphocytic infiltrate. Extravasation of red blood cells and basal layer vacuolar change.

Histological description showed parakeratosis and mild acanthosis in the epidermis layer, and perivascular lymphocytic infiltrates in the papillary dermis, extravasation of red blood cells, and basal layer vacuolar change were also observed. Histological findings were consistent with PLC (Figure 2).

Autoimmunity tests revealed a positive rheumatoid factor in 148 UI/ml (<14 UI/ml) and the antinuclear antibodies (ANAs) were negative.

Treatment with daily topical corticosteroids was initiated. After one month of treatment, the rash has decreased and treatment with etanercept was continued.

## 3. Discussion

The tumor necrosis factor alpha (TNF-*α*) antagonists are widely used for the treatment of chronic inflammatory disorders including RA. The cutaneous adverse reactions are an important component described in this therapeutic group [5]. Hernández et al. analyzed the incidence rate of cutaneous adverse events in patients with chronic inflammatory rheumatic diseases treated with TNF-*α* antagonists. A total of 5,437 patients were included with 920 cutaneous adverse events reported without descriptions of PLC [6].

The etanercept is a recombinant fusion protein with two human extracellular domains linked to an IgG1 Fc domain, which inhibits the link of TNF-*α* with his receptor [7]. The injection site reactions are reported to be the most common adverse event of etanercept therapy [8]; however, etanercept is associated with other wide varieties of dermatologic adverse events different to injection site reactions, among which are cutaneous infections, immune-mediated complications such as psoriasis, cutaneous lupus, cutaneous vasculitis, and malignant neoplasms [9, 10]. There are no reported cases in the literature of PLC associated with etanercept therapy.

The PLC is a rare inflammatory skin disorder of unknown etiology that is considered a subtype of pityriasis lichenoides. Patients with PLC usually present with widely distributed red-brown papules on the trunk and extremities, and diagnosis of PLC is suspected based on the findings of the physical examination. A skin biopsy is usually performed to confirm the diagnosis [1].

The appearance of PLC has been described with the use of anti-TNF therapy, two cases in patients with Crohn's disease under treatment with adalimumab [2] and two case reports with infliximab in a patient with psoriasis and another case with spondyloarthritis associated with ulcerative colitis [3, 4]. But to our knowledge, this is the first case report of PLC related to etanercept. Data on the efficacy of treatments for PLC are limited. Our patient showed improvement with the use of topical steroids, and the treatment with etanercept was continued with remission of RA.
